# Predicting Phenoconversion in Isolated RBD: Machine Learning and Explainable AI Approach

**DOI:** 10.3390/clockssleep7020019

**Published:** 2025-04-11

**Authors:** Yong-Woo Shin, Jung-Ick Byun, Jun-Sang Sunwoo, Chae-Seo Rhee, Jung-Hwan Shin, Han-Joon Kim, Ki-Young Jung

**Affiliations:** 1 Department of Neurology, Inha University Hospital, Incheon 22332, Republic of Korea; reervlftmd@inha.ac.kr; 2 Department of Neurology, Kyung Hee University Hospital at Gangdong, Seoul 05278, Republic of Korea; jbyun@khu.ac.kr; 3 Department of Neurology, Kangbuk Samsung Hospital, Seoul 03181, Republic of Korea; ultrajs4@gmail.com; 4 Department of Otorhinolaryngology-Head and Neck Surgery, Seoul National University Hospital, Seoul National University College of Medicine, Seoul 03080, Republic of Korea; 5 Department of Neurology, Seoul National University Hospital, Seoul National University College of Medicine, Seoul 03080, Republic of Korea; neo2003@snu.ac.kr

**Keywords:** REM sleep behavior disorder, machine learning, phenoconversion, neurodegenerative disorders, Parkinson’s disease, survival analysis

## Abstract

Isolated rapid eye movement (REM) sleep behavior disorder (iRBD) is recognized as a precursor to neurodegenerative diseases. This study aimed to develop predictive models for the timing and subtype of phenoconversion in iRBD. We analyzed comprehensive clinical data from 178 individuals with iRBD over a median follow-up of 3.6 years and applied machine learning models to predict when phenoconversion would occur and whether progression would present with motor- or cognition-first symptoms. During follow-up, 30 patients developed a neurodegenerative disorder, and the extreme gradient boosting survival embeddings–Kaplan neighbors (XGBSE-KN) model demonstrated the best performance for timing (concordance index: 0.823; integrated Brier score: 0.123). Age, antidepressant use, and Movement Disorder Society–Unified Parkinson’s Disease Rating Scale Part III scores correlated with higher phenoconversion risk, while coffee consumption was protective. For subtype classification, the RandomForestClassifier achieved the highest performance (Matthews correlation coefficient: 0.697), indicating that higher Montreal Cognitive Assessment scores and younger age predicted motor-first progression, whereas longer total sleep time was associated with cognition-first outcomes. These findings highlight the utility of machine learning in guiding prognosis and tailored interventions for iRBD. Future research should include additional biomarkers, extend follow-up, and validate these models in external cohorts to ensure generalizability.

## 1. Introduction

Isolated rapid eye movement (REM) behavior disorder (iRBD) is an early indicator of alpha-synuclein-mediated neurodegenerative diseases such as Parkinson’s disease (PD), dementia with Lewy bodies (DLB), and multiple system atrophy (MSA) [1,2,3,4]. More than 90% of patients with iRBD ultimately convert to overt synucleinopathies within 14 years of diagnosis [5]. This high conversion rate suggests the importance of iRBD as a prodromal stage for alpha-synuclein-mediated neurodegenerative diseases.

The timing of phenoconversion ranges from months to decades. Longitudinal studies have identified several risk factors, including advanced age, hyposmia, color vision abnormalities, mild parkinsonism, cognitive decline, autonomic disturbances, nigrostriatal dopaminergic impairment, and REM sleep without atonia loss [6,7,8]. While these findings help identify high-risk iRBD patients, they focused on identifying general risk factors rather than developing personalized prediction models [2,4,5,6,7]. As a result, successful translation into personalized, actionable prediction models for clinical use has not been achieved.

Prognostic counseling in iRBD patients is indispensable for managing future health risks, enabling early interventions, and providing psychological support needed. A previous study found that most patients had a strong preference for detailed prognostic information [9]. However, it is often challenging to discuss prognostication, given the uncertainty in accurate personalized prediction, and the lack of a standardized approach [10,11]. This highlights the urgent need for tools that can support prognostication.

To address these issues, recent attempts have been made utilizing machine learning (ML) approaches to predict prognosis in iRBD patients. For instance, one study employed ML models using baseline electroencephalography (EEG) features to predict phenoconversion time and subtype in iRBD patients [12]. Another study developed a fully automated ML framework using polysomnography (PSG) data to predict phenoconversion, highlighting the potential of ML in this domain [13]. Both studies, while demonstrating the potential of ML, relied solely on a random survival forest model and a single feature selection method, without exploring alternative models or optimizing predictors. Furthermore, their reliance on specialized equipment like EEG and PSG limits practicality in routine clinical settings, leaving room for improvement in identifying more accessible and effective approaches.

In this study, we aimed to address these limitations by developing machine learning models to predict phenoconversion time and subtype in iRBD using readily available clinical indicators. This approach enhances practicality for routine clinical use while facilitating early identification of high-risk patients.

## 2. Results

### 2.1. Study Population

**Figure 1:**
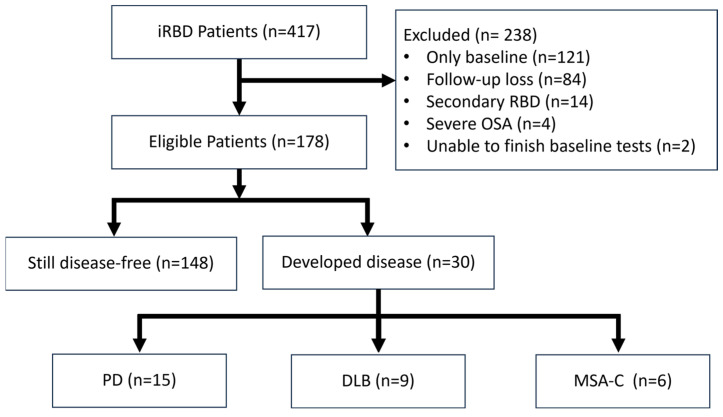
Flow-chart of patient selection.

Out of 417 registered patients, 178 were eligible for analysis (Figure 1). During a median follow-up of 3.6 years, 30 participants developed a neurodegenerative disease. At two years follow-up, 4.8% had a risk of developing neurodegenerative diseases, which rose to 18.3% at four years and 23.3% at 6 years (Supplementary Figure S1). Among the patients phenoconverted, PD was the most frequent diagnosis, with about 50% of cases (15 patients). This was followed by DLB, representing 30% of conversions (9 patients), and Multiple System Atrophy-Cerebellar type (MSA-C), comprising 20% (6 patients). The remaining 148 patients showed no evidence of neurodegenerative disease during the study period and were categorized as still disease-free.

### 2.2. Clinical and Demographic Characteristics

Each prodromal marker was analyzed as either a continuous or categorical variable, as appropriate. The Cox Proportional Hazards (CoxPH) model was used to calculate the hazard ratio (HR) for each variable. This approach was employed for both unadjusted and age- and sex-adjusted analyses, if applicable. Several factors emerged as significant predictors of phenoconversion (Table 1), including age, with an HR of 1.09 (95% CI [1.03–1.15]); use of antidepressants, HR 3.69 (95% CI [1.62–8.44]); solvent exposure, HR 2.73 (95% CI [1.24–5.99]); and coffee use, HR 0.42 (95% CI [0.20–0.90]). Furthermore, components of the Pittsburgh Sleep Quality Index, specifically PSQI-C3 (sleep duration) and PSQI-C4 (sleep efficiency), yielded HRs of 0.61 (95% CI [0.41–0.92]) and 0.64 (95% CI [0.44–0.92]), respectively. MDS-UPDRS-III excluding action and postural tremor scores was also significant, with an HR of 1.32 (95% CI [1.07–1.63]). Additionally, a multivariate Cox regression analysis was performed using significant variables from the univariate analysis (see Supplementary Table S1).

We analyzed the characteristics of iRBD patients based on the subtype of phenoconversion: cognition-first (*n* = 9) and motor-first (*n* = 21). There were significant differences between the groups (Table 2). The cognition-first group was significantly older (cognition-first: 75.0 years versus motor-first: 67.0 years; *p* = 0.02). Different performances on cognitive assessments were also noticed; those in the cognition-first group scored lower on both MMSE (cognition-first: 26.0 versus motor-first: 28.0; *p* = 0.01) and MoCA (cognition-first: 23.0 versus motor-first: 26.0; *p* = 0.03). Furthermore, an intriguing difference was observed in the total sleep time component of the PSQI (PSQI-TST). The cognition-first group had significantly longer sleep durations compared to the motor-first group (8.4 h versus 7.0 h, respectively; *p* = 0.03).

### 2.3. Phenoconversion Time Prediction

The evaluation of the phenoconversion prediction models was conducted using four feature selection methods, and 10 models with 10-fold cross-validation. In our analysis of the phenoconversion time prediction models, the extreme gradient boosting survival embeddings–Kaplan neighbors (XGBSE-KN) model [14], featuring predictors selected by Recursive Feature Elimination with Random Survival Forest (RFE-RSF) [15,16], emerged as the most effective, achieving an IBS of 0.123 (±0.03) and a C-index of 0.823 (±0.14), the latter indicating a high degree of accuracy in predicting the correct sequence of patient phenoconversion events (Supplementary Figures S2 and S3). The final features selected by RFE-RSF included weight, MDS-UPDRS III excluding tremor, antidepressant use, RBDQ-KR factor 2 (behavioral manifestation), coffee use, and age (Supplementary Table S2).

SHAP (SHapley Additive exPlanations) analysis provided useful information on the relationships between features and phenoconversion risk. When evaluating the mean absolute SHAP values, which indicate the overall impact of each feature on the model’s output, age was found to be the most influential factor (Supplementary Figure S4). This was followed, in order of importance, by RBDQ-KR factor 2, weight, antidepressant use, coffee use, and MDS-UPDRS III excluding tremor (Supplementary Figure S4). These results highlight the significant role of age as a predictor in the model and the varying degrees of influence of other factors. Analysis of SHAP values (Supplementary Figure S5) revealed both monotonic and non-linear relationships between features and phenoconversion risk. Specifically, age, antidepressant use, and MDS-UPDRS III excluding tremor showed generally positive monotonic relationships with risk, while coffee use showed a protective monotonic relationship (negative correlation). In contrast, body weight and RBDQ-KR factor 2 exhibited non-linear relationships. For instance, the risk associated with weight increased notably within the 55–65 kg range. Similarly, RBDQ-KR Factor 2 displayed a complex pattern, with an elevated risk for scores below 20 and again for scores between 35 and 45. Notably, the features exhibiting monotonic relationships in the SHAP analysis (age, antidepressant use, MDS-UPDRS III excluding tremor, coffee use) corresponded to those found significant in the initial univariable analysis. Conversely, the features with non-linear SHAP relationships (body weight, RBDQ-KR factor 2) were not identified as significant factors in the univariable analysis.

### 2.4. Phenoconversion Subtype Prediction

In addition to developing a model for predicting the time to phenoconversion, we also focused on distinguishing the subtype of phenoconversion—specifically, whether patients would exhibit a motor-first or cognition-first progression. We identified several clinical indicators that were significantly different between motor-first and cognition-first in a univariable analysis, including age, MMSE, MoCA, and the PSQI-TST. In assessing MIC values, PSQI-TST and MMSE showed significantly high contributions (Supplementary Table S3). We then utilized the mRMR method for optimal feature selection resulting in PSQI-TST, MoCA, and age. Additionally, the Boruta algorithm was implemented, affirming the same set of features as identified by mRMR.

We developed ten classifiers to predict phenoconversion subtypes, utilizing both a univariable feature set and mRMR feature set. To enhance the classifiers’ performance, we applied SMOTE and Self-Training methods. Among these, the RandomForestClassifier (RF) showed the best performance across both feature sets, particularly with mRMR, achieving the highest MCC value of 0.697 through 100-repeated 5-fold stratified cross-validation (Supplementary Table S4, Supplementary Figures S6 and S7). RF also excelled in metrics like macro F1, accuracy, precision, recall, balanced accuracy, and Cohen’s Kappa, as well as excelling in the AUC specifically calculated using LPOCV, demonstrating its robustness (Supplementary Table S4).

When using RF and mRFR feature sets to obtain SHAP values, PSQI-TST was the most important, followed by MoCA and age (Supplementary Figure S8). A PSQI-TST of more than 8 h contributed to a cognition-first prediction, while an MoCA score of 25 or higher and age of 70 or younger contributed to a motor-first prediction (Supplementary Figure S9).

### 2.5. Web Deployment

**Figure 2:**
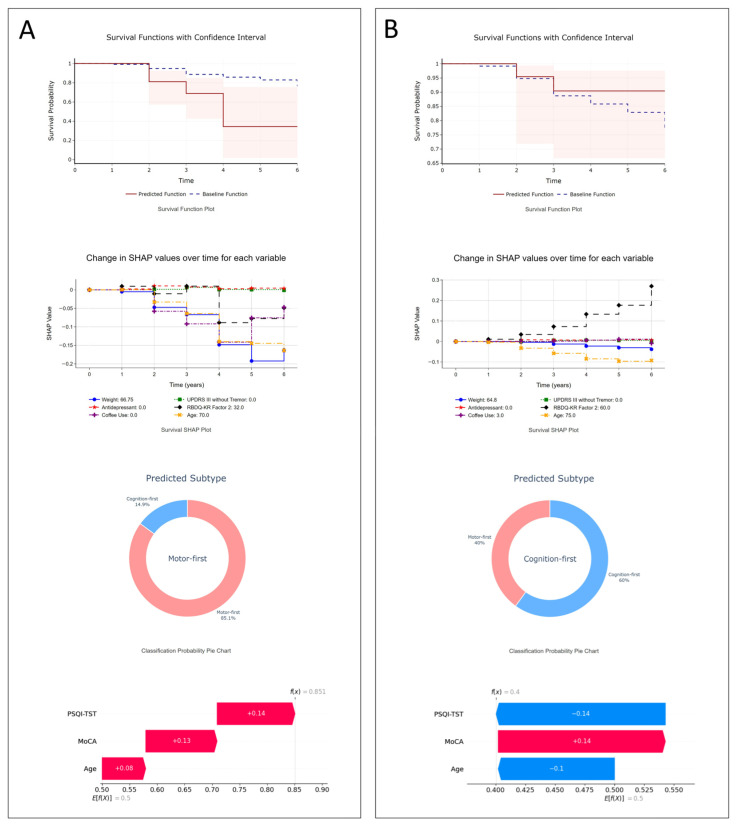
Illustrative examples: (**A**) A 70-year-old female with dream enactment behaviors since age 63, initially MDS-UPDRS-III = 0, MoCA-K = 28, mild depression. After 3.9 years, she developed Parkinson’s disease. The model projects a steep drop in phenoconversion-free survival (34% by the fourth year). SHAP points to age, lack of antidepressant use, and other factors as contributors to her rapid motor-first phenoconversion, confirmed by clinical outcomes; (**B**) a 75-year-old male with dream enactment behaviors for three years before RBD diagnosis, initial MDS-UPDRS III = 0, MoCA-K = 25, and decreased smell (KVSS2-16). Six years later, he remains neurodegenerative disease-free. The model predicts a 90% phenoconversion-free probability at six years; SurvSHAP highlights age as a risk factor and RBDQ-KR Factor 2 as protective. His subtype prediction leans cognition-first, with age and PSQI-TST identified by SHAP as key factors.

A user-friendly web application was developed for physician access, which included both models: the XGBSE-KN model for phenoconversion time and the RF model for subtype prediction. The application offers individualized predictions and rationales based on input characteristics, aiding physicians in making informed decisions about patient management and prognosis (Supplementary Figure S10). To demonstrate the use case of the model, we generated a report using actual patient cases, which is shown in Figure 2.

## 3. Discussion

In this study, we developed machine learning models to predict phenoconversion time and subtype in patients with iRBD. Leveraging a patient cohort with comprehensive clinical markers, our findings identified key predictors of phenoconversion and contributed to the reliability of the generated model.

The XGBSE-KN model, using features selected through the RFE-RSF method, was the most effective model for phenoconversion time prediction, achieving an excellent concordance index of 0.823 and a good IBS of 0.123. For subtype classification, the RF demonstrated the highest performance, with a good to excellent MCC of 0.697. These results underscore the potential of advanced machine learning techniques in accurately predicting the prognosis in patients with iRBD.

Previous studies using EEG and PSG reported C-indices of 0.775 and 0.723~0.741, respectively, with IBS values of 0.114 and 0.174 [12,13]. While direct comparisons are challenging due to differences in cohorts and modalities, our results demonstrate that clinical indicators alone can achieve comparable or even higher performance. Future studies could explore integrating advanced diagnostic features, such as EEG or PSG, with clinical data to further enhance prediction accuracy.

Our research encompassed a cohort of 178 patients with iRBD, utilizing an extensive array of clinical variables to enhance the study’s reliability and value. Over a median follow-up duration of 3.6 years, 30 individuals progressed to a neurodegenerative condition. Our study identified several critical predictors of phenoconversion time, including age, antidepressant use, solvent exposure, and coffee consumption. Additionally, components of the PSQI, PSQI-C3 (sleep duration), and PSQI-C4 (sleep efficiency), and MDS-UPDRS part III excluding tremor scores, were found to be significant.

Old age was identified as a significant predictor, which aligns with existing research suggesting a higher vulnerability to neurodegenerative diseases as one ages. Solvent exposure and antidepressant use were identified as significant risk factors for phenoconversion, while coffee consumption showed a protective effect. These findings align with existing research on Parkinson’s disease (PD), though their relationship with other synucleinopathies like MSA and DLB is less clear [17,18,19,20,21,22,23]. In our study, we also found that antidepressant use at the baseline evaluation was a significant risk factor for phenoconversion in patients with iRBD. Antidepressant use has been associated with symptoms of RBD. Patients with iRBD who take antidepressants show significant abnormalities in neurodegenerative markers, such as olfaction, color vision, and motor function. A previous study using 18F-DOPA showed that 18F-DOPA uptake was significantly lower in patients with co-morbid RBD and major depressive disorder (MDD) compared to patients taking medication for MDD. This suggests that the development of RBD symptoms in MDD patients is not simply an antidepressant-induced condition, but an early stage of α-synucleinopathy [24]. In addition, it is well known that depression is a pre-motor symptom of PD, along with iRBD, anosmia, and constipation [25,26]. Thus, the relationship between depression, antidepressant use, and iRBD is complex and may reflect an underlying neurodegenerative process rather than a simple medication effect. Additionally, while weight and RBD-KR Factor 2 did not emerge as statistically significant in traditional analyses, they were selected in the final model and exhibited complex relationships when assessed via SHAP values. It is difficult to determine the direct mechanistic link between weight, RBD-KR Factor 2, and phenoconversion, suggesting the need for further research. They may not directly contribute to neurodegeneration but could be indirectly related to phenoconversion risk by reflecting changes in other covariates.

Selected features for the final model were slightly different from the features identified with statistical methods. Age, antidepressant use, MDS-UPDRS III excluding tremor, and coffee use were identified in the univariable analysis and the SHAP analysis revealed the linear relationship with the phenoconversion risk. On the other hand, RBDQ-KR Factor 2 and weight were not identified with the statistical methods and SHAP analysis revealed non-linear relationships with the phenoconversion risk. This illustrates the capability of machine learning models to uncover intricate relationships that traditional statistical methods might overlook, especially in large variable sets. It might be possible to identify these non-linear relationships by using strategies like stratifying the variables, but it would not be easy to find these relationships among many variables without missing them. We also note that several factors significant in the univariable analysis, such as education, solvent exposure, MMSE, MoCA, PSQI-C3, and PSQI-TST, were not retained in the final ML model’s feature set. This exclusion might occur because the predictive information contained in these variables was potentially redundant or already captured by other selected features; for instance, age itself correlates with cognitive scores like MMSE and MoCA, or the effect of solvent exposure might be indirectly represented.

Our study highlights limitations of traditional survival analysis methods like CoxPH, which struggle with non-linear interactions and high-dimensional data. Despite using CoxNet, an extension of the CoxPH model that integrates L1 and L2 regularization to mitigate these limitations, performance remained poor, highlighting the need for advanced machine learning techniques in analyzing complex clinical data. Consequently, we developed models like XGBSE-KN, which demonstrated markedly superior predictive accuracy. While acknowledging that such sophisticated models demand more computation and pose greater interpretability challenges than traditional methods, we consider this a justifiable trade-off for the enhanced performance needed for personalized medicine. Encouragingly, our experimental web application suggested feasible resource requirements for potential deployment, and SHAP analyses provided partial model interpretability, mitigating some ‘black box’ concerns. Thus, despite inherent complexities, advanced ML techniques appear essential for analyzing intricate clinical data.

In examining the subtypes of phenoconversion, cognition-first patients had lower baseline MMSE and MoCA scores, which was confirmed by previous studies [6]. Interestingly, the PSQI-TST was longer in the cognition-first group, contrasting with previous PSG findings in DLB patients that reported lower sleep efficiency, total sleep time, and REM sleep duration, and higher sleep latency and wake after sleep onset (WASO) [27]. A possible explanation for the increased PSQI-TST observed in our study is that patients might not exclude periods of WASO, thereby reporting longer sleep durations in the PSQI-TST—even though their effective sleep time might be reduced due to frequent awakenings or disturbances. This is supported by a previous large study comparing PSG, actigraphy, and sleep diaries, which found that sleep diaries tend to underestimate sleep latency and WASO, and exaggerate time in bed, total sleep time, and sleep efficiency [28]. Furthermore, according to a systematic review and meta-analysis, TST in all-cause cognitive decline or dementia exhibits a U-shaped relationship, with both short and long sleep durations associated with an increased risk of cognitive decline or dementia [29]. Although this study was not specific to DLB, the finding that longer sleep duration is linked to cognitive decline suggests that the extended TST in our cognition-first group may be indicative of underlying neurodegenerative processes [29]. We found that the cognition-first group was older than the motor-first group at the baseline. This finding is consistent with established epidemiological patterns in synucleinopathies. PD incidence increases markedly after age 60, with a mean diagnosis age of 70.5 years [30]. MSA, also in the motor-first category, presents earlier, with onset typically between 54 and 58 years [31,32,33]. Conversely, DLB, often associated with cognitive-first presentation, shows peak incidence beyond age 70 [34,35]. Our cohort reflected these patterns, with the motor-first group’s median age at 67.0 years and the cognition-first group at 75.0 years. The SHAP dependency plot in our prediction model was in good agreement with these findings, displaying a notable value shift around age 70.

To capture non-linear relationships between subtypes and variables, we utilized MIC. However, in subtype classification, it was hard to find such non-linear features. This could be due to small sample sizes or the absence of non-linear relationships in our clinical metrics. Additionally, we developed a user-friendly web application for physicians, demonstrating the potential of machine learning in providing more accurate and personalized insights in medical practice.

Our study has several important limitations. Firstly, the generalizability of our findings is limited by the fact that this was a single-center study without external validation. Secondly, with only 30 phenoconverters among 178 participants during the 3.6-year follow-up, the relatively small sample size and short duration may not fully capture the spectrum of phenoconversion patterns, potentially biasing results toward early converters. The limited number of cases also affected subtype prediction, necessitating broad categorization into “motor-first” and “cognition-first” groups, including only six patients with MSA. Furthermore, while we collected comprehensive clinical data, certain potential factors such as detailed neuropsychological assessments and various environmental factors were not included. Additionally, we could not adopt more rigorous internal validation strategies, largely due to concerns about computational complexity and our limited sample size. As a result, there is an increased risk of overfitting, and our model performance estimates may be overly optimistic.

Consequently, this study should be considered exploratory, and the models are not yet ready for direct application in routine clinical practice. While it successfully demonstrates the potential of using machine learning with clinical predictors for iRBD phenoconversion, the developed models require rigorous external validation in larger, diverse cohorts before their predictive accuracy and clinical utility can be established. Incorporating additional biomarkers and extending follow-up durations could further enhance performance and applicability.

In conclusion, this study demonstrates the potential of using machine learning with clinical indicators to predict phenoconversion timing and subtype in iRBD patients. These tools offer significant promise for improving personalized risk assessments and clinical decision-making. However, external validation and further research in diverse populations are essential to fully realize their clinical impact.

## 4. Materials and Methods

### 4.1. Study Population and Data Collection

Patients were recruited from the iRBD registry at the Seoul National University Hospital from April 2016 to May 2022. Participants underwent diagnostic overnight video-polysomnography (vPSG) and were enrolled upon meeting International Classification of Sleep Disorders, 3rd edition criteria for iRBD. At recruitment, two neurologists specializing in sleep medicine (JK) and movement disorders (KH) performed comprehensive evaluations to exclude secondary causes of RBD, comorbid neurodegenerative diseases, and major medical illnesses.

Subjects with any preexisting neurodegenerative diseases were excluded from the registry. In addition, the study’s exclusion criteria also included participants with a history of neurological disorders like epilepsy or stroke, past psychiatric illness, head trauma, and the recent use of medications known to affect sleep or motor functions. Another key exclusion factor was severe obstructive sleep apnea, defined as having an apnea–hypopnea index of 30 or higher on baseline vPSG. Moreover, individuals with serious medical comorbidities were also excluded. All subjects satisfying all the eligibility criteria provided written informed consent before being included in the study, which was approved by the Institutional Review Board of the Seoul National University Hospital (IRB No.: 1708-169-883, 1507-100-689).

### 4.2. Clinical Evaluation

Comprehensive demographic, medical history and clinical evaluation data were collected from all the participants. Demographic variables included age, sex, height, weight, and body mass index (BMI). Past medical history included medical illness, psychiatric illness, use of antidepressants, alcohol consumption, smoking, history of pesticide exposure or solvent exposure, and PD in first-degree relatives. Clinical assessments evaluated olfactory loss, prior injury, daily coffee consumption, and years of education. We assessed olfactory loss using the Korean Version of the Sniffin’ Sticks test (KVSS) [36]. The frequency and severity of RBD symptoms were measured using the Korean version of REM Sleep Behavior Disorder Questionnaire-Hong Kong (RBDQ-KR) [37]. Cognitive functions were measured with the Mini-Mental State Exam (MMSE), Montreal Cognitive Assessment (MoCA) [38,39]. Sleep quality and daytime sleepiness assessments included the Epworth Sleepiness Scale (ESS), the Insomnia Severity Index (ISI), and the Pittsburgh Sleep Quality Index (PSQI) [40,41,42]. We evaluated the mental health of the participants by the Geriatric Depression Scale (GDS) [43]. Autonomic dysfunction was quantified by the SCOPA-AUT scale including gastrointestinal, urinary, cardiovascular, and sexual domains [44]. Motor performance was graded with the Movement Disorders Society–Unified Parkinson’s Disease Rating Scale Part III (MDS-UPDRS-III) [45]. Phenoconversion in iRBD patients was assessed every 6 to 12 months by the same two neurologists (JK and KH). The diagnoses of PD, DLB, and MSA were made according to standard criteria [46,47,48].

### 4.3. Data Preparation and Imputation for Model Predictors

Variables with greater than 30 percent missing values were excluded. For the remaining variables, multiple imputations by chained equations were used. Nominal variables were encoded using one-hot or binary encoding, while ordinal variables were label-encoded. Only baseline measurements were used as model predictors.

### 4.4. Model Development—Prediction of Phenoconversion Time

Four feature selection techniques were applied: univariate filtering, L1 regularization, recursive feature elimination (RFE), and SelectKBest (SKB). Performance was measured by concordance index (C-index) and integrated Brier score (IBS) [49,50]. The C-index reflects how well the model ranks the survival times, while the IBS measures the calibration and refinement of the survival probabilities.

Survival analysis machine learning models, including elastic net, accelerated failure time models, random survival forests, survival tree, gradient boosting machines (GBMs), and extreme gradient boosting survival embeddings (XGBSEs), were developed and evaluated using 10-fold cross-validation [14,15,51,52,53,54,55,56]. Hyperparameter optimization utilized Bayesian optimization [57]. To compare the performance of the developed machine learning models in survival analysis, we adopted the corrected resampled paired t-test method as proposed by Nadeau and Bengio [58]. For multiple testing correction, we incorporated the Benjamini–Hochberg (BH) procedure. After identifying the optimal feature selection method and model architecture through cross-validation, the final model was trained using the full dataset to maximize predictive performance.

All analyses were conducted using Python 3.10.9, with key libraries including scikit-learn (v.1.1.3), scikit-survival (v.0.19.0), lifelines (v.0.27.4), optuna (v.3.2.0), pandas (v1.5.3), numpy (v1.22.3), and xgboost-survival-embeddings or XGBSE (v0.2.3). Additionally, matplotlib (v3.7.1) and seaborn (v0.11.2) were used for data visualization.

### 4.5. Model Development—Prediction of Phenoconversion Subtype

We also developed predictive models for the subtype of phenoconversion in iRBD, differentiating between “motor-first” subtypes, such as PD and MSA, and the “cognition-first” subtype, exclusively DLB. We applied univariable analysis for initial feature selection to identify predictors differentiating between “motor-first” and “cognition-first” subtypes. To capture non-linear relationships, we utilized the Minimum redundancy maximum relevance (mRMR) feature selection method [59,60]. We developed eleven classifiers using various machine learning algorithms. To tackle the challenges of the small and imbalanced dataset in our RBD subtype prediction model, we integrated the Synthetic Minority Over-sampling Technique (SMOTE) and the Self-Training method, a semi-supervised learning approach [61,62]. SMOTE was used to balance the class distribution, while Self-Training leveraged unlabeled data to augment the training dataset, enhancing the overall effectiveness of our predictive models. Due to the small and imbalanced dataset comprising only 30 patients in our RBD subtype prediction model, we employed a 100-repeated 5-fold stratified cross-validation to ensure robustness and reliability in our evaluation. This approach was chosen over a 10-fold CV to obtain more stable and reliable estimates by increasing the number of repetitions and ensuring that each data point was adequately represented in both the training and validation sets. For statistical analysis, we applied the correction method by Bouckaert and Frank [63] and used the R library CorrectR [64]. For multiple testing correction, we incorporated the Benjamini–Hochberg (BH) procedure.

The Matthews correlation coefficient (MCC) was chosen as the primary evaluation metric because of its robustness for binary classification, especially with imbalanced class distributions [65,66,67]. MCC scores range from −1 to 1, with 1 indicating perfect prediction, 0 representing random guessing, and −1 meaning total disagreement. Along with the MCC, several secondary performance metrics were also calculated, including F1 score, precision, recall, and accuracy. Separately, the Receiver Operating Characteristic Area Under the Curve (ROC-AUC) was calculated using a leave-pair-out cross-validation approach (LPOCV). This method was specifically chosen for its ability to minimize bias in the evaluation of the AUC, particularly important in studies with smaller sample sizes [68]. Upon identifying the optimal feature set and classifier architecture, the final classification model was trained with the complete dataset to maximize prediction accuracy.

All analyses were conducted using Python 3.10.9, with key libraries including scikit-learn (v.1.1.3), xgboost (v1.7.5), optuna (v.3.2.0), pandas (v1.5.3), and numpy (v1.22.3). Additionally, matplotlib (v3.7.1), and seaborn (v0.11.2) were used for data visualization.

### 4.6. Model Explanation and Deployment

For the phenoconversion time prediction model, Kernel SHAP was employed to interpret the contributions of individual features [69]. Additionally, we incorporated SurvSHAP, a method for time-varying feature importance in survival models, which allowed for a more dynamic understanding of how different variables influence the risk of phenoconversion over time [70]. All SHAP values were calculated using the final models trained on the full dataset, ensuring comprehensive interpretation of feature importance across the entire study population. The developed model was deployed as a web application to facilitate ease of use for clinicians. An overview of the study design and patient selection are presented in Supplementary Figure S11.

## Figures and Tables

**Table 1:** Baseline predictors for neurodegenerative phenoconversion in iRBD.

Variable		Developed Disease (*n* = 30)	Still Disease-Free (*n* = 148)	HR, Unadjusted (95%CI)	HR, Adjusted (95% CI)
Age		69.5 (65.0–74.0)	64.0 (60.0–70.25)	1.09 (1.03–1.15)	1.09 (1.03–1.15) *
Sex, % male		56.7%	63.5%	0.70 (0.34–1.45)	0.78 (0.38–1.61) *
Height, cm		163.1 (156.8–166.8)	165.0 (158.0–170.0)	0.97 (0.94–1.01)	1.00 (0.94–1.07)
Weight, kg		62.0 (56.2–67.7)	64.0 (59.0–71.0)	0.97 (0.94–1.01)	0.99 (0.94–1.03)
BMI, kg/m^2^		23.5 (22.4–24.7)	24.4 (22.5–25.7)	0.95 (0.83–1.08)	0.95 (0.83–1.08)
RLS		6.7%	6.1%	0.97 (0.23–4.09)	1.05 (0.25–4.43)
Diabetes		16.7%	16.2%	1.09 (0.42–2.85)	0.94 (0.36–2.48)
Melatonin		56.7%	64.9%	0.59 (0.28–1.23)	0.47 (0.22–1.00)
Antidepressants		26.7%	7.4%	3.60 (1.60–8.11)	3.69 (1.62–8.44)
Alcohol use				0.74 (0.50–1.10)	0.75 (0.49–1.15)
	Non-drinker	66.7%	50.7%		
	1–2 times/month	10.0%	23.0%		
	1–2 times/week	20.0%	17.6%		
	3–4 times/week	3.3%	6.8%		
	Daily	0.0%	2.0%		
Smoking					
	Never	66.7%	51.4%	1	1
	Current	6.7%	10.1%	0.47 (0.11–2.03)	0.68 (0.14–3.25)
	Former	26.7%	38.5%	0.54 (0.24–1.24)	0.49 (0.18–1.32)
Pesticide exposure		20.0%	14.2%	1.67 (0.68–4.10)	1.41 (0.56–3.55)
Solvent exposure		30.0%	14.2%	2.66 (1.22–5.82)	2.73 (1.24–5.99)
Olfactory loss †		53.3%	60.8%	0.90 (0.44–1.85)	0.64 (0.29–1.42)
Injury				0.86 (0.47–1.58)	0.83 (0.45–1.52)
	None	63.3%	57.4%		
	Minor	30.0%	35.8%		
	Major	6.7%	6.8%		
Coffee use ‡		63.3%	83.1%	0.43 (0.21–0.91)	0.42 (0.20–0.90)
Daily coffee consumption ‡		1.0 (0.0–2.0)	1.0 (1.0–2.0)	0.83 (0.60–1.15)	0.91 (0.63–1.32)
DEB frequency		3.0 (2.0–6.5)	3.0 (1.0–7.0)	0.99 (0.86–1.14)	1.00 (0.87–1.16)
Low education §		26.7%	15.5%	1.92 (0.85–4.31)	1.57 (0.58–4.21)
Education				0.73 (0.56–0.96)	0.80 (0.59–1.09)
	No education	3.3%	1.4%		
	Elementary school (6 years)	23.3%	14.2%		
	Middle school (9 years)	20.0%	9.5%		
	High school (12 years)	26.7%	26.4%		
	Bachelor’s degree (16 years)	20.0%	39.9%		
	Above bachelor’s degree (18 years)	6.7%	8.8%		
First-degree relative with PD		6.7%	10.1%	0.71 (0.17–2.98)	0.75 (0.17–3.22)
RBDQ-KR		50.0 (38.2–55.8)	48.0 (36.0–61.0)	0.99 (0.97–1.01)	0.99 (0.97–1.01)
	RBDQ–KR Factor 1	10.5 (8.0–19.0)	13.0 (9.0–16.0)	0.99 (0.93–1.05)	0.99 (0.93–1.05)
	RBDQ–KR Factor 2	35.5 (28.5–42.2)	35.0 (26.0–45.0)	0.98 (0.96–1.01)	0.98 (0.96–1.01)
MMSE		27.0 (26.0–28.0)	28.0 (27.0–29.0)	0.85 (0.76–0.96)	0.90 (0.79–1.03)
MoCA		24.0 (22.2–26.8)	26.0 (24.0–29.0)	0.93 (0.87–0.99)	0.95 (0.88–1.03)
Epworth Sleepiness Scale		5.0 (3.2–7.0)	5.0 (3.0–8.0)	1.01 (0.92–1.11)	1.01 (0.92–1.11)
K-GDS		11.0 (4.5–16.0)	8.0 (4.0–14.0)	1.02 (0.97–1.07)	1.01 (0.96–1.06)
Insomnia Severity Index		7.0 (4.5–11.5)	6.0 (3.0–12.0)	1.01 (0.96–1.07)	1.00 (0.94–1.05)
PSQI		5.0 (3.0–7.8)	6.0 (4.0–9.0)	0.96 (0.87–1.05)	0.94 (0.86–1.03)
	TST	7.3 (6.5–8.0)	7.0 (6.3–7.7)	1.53 (1.12–2.08)	1.34 (0.98–1.83)
	C1	2.0 (1.0–2.0)	1.0 (1.0–2.0)	1.28 (0.86–1.92)	1.19 (0.78–1.79)
	C2	0.5 (0.0–1.0)	1.0 (0.0–2.0)	0.76 (0.50–1.14)	0.67 (0.44–1.01)
	C3	0.0 (0.0–1.0)	1.0 (0.0–2.0)	0.59 (0.38–0.92)	0.61 (0.40–0.92)
	C4	0.0 (0.0–0.8)	0.0 (0.0–2.0)	0.76 (0.52–1.09)	0.64 (0.44–0.92)
	C5	1.0 (1.0–1.0)	1.0 (1.0–1.2)	1.10 (0.55–2.21)	0.91 (0.45–1.82)
	C6	0.0 (0.0–0.0)	0.0 (0.0–0.0)	1.09 (0.80–1.48)	1.13 (0.82–1.54)
	C7	1.0 (0.0–1.0)	0.5 (0.0–1.0)	1.09 (0.73–1.63)	1.21 (0.82–1.77)
SCOPA-AUT total		12.5 (6.5-19.8)	10.0 (5.0–16.0)	1.03 (0.99–1.08)	1.02 (0.98–1.07)
SCOPA-AUT Orthostatic hypotension ¶					
	No	56.7%	66.2%	1	1
	Borderline	36.7%	27.0%	1.34 (0.64–2.82)	1.32 (0.63–2.78)
	Yes	6.7%	6.8%	0.71 (0.17–3.00)	0.68 (0.16–2.90)
SCOPA-AUT Constipation ‖					
	No	26.7%	33.8%	1	1
	Borderline	46.7%	39.2%	1.57 (0.66–3.75)	1.45 (0.58–3.62)
	Yes	26.7%	27.0%	1.29 (0.48–3.43)	1.13 (0.42–3.07)
SCOPA-AUT Urinary ‖					
	No	3.3%	14.2%	1	1
	Borderline	50.0%	52.0%	4.46 (0.59–33.77)	4.41 (0.58–33.37)
	Yes	46.7%	33.8%	5.69 (0.75–43.32)	4.96 (0.65–37.93)
SCOPA-AUT Erectile Dysfunction ‖					
	No	10.0%	18.9%	1	1
	Borderline	23.3%	22.3%	2.02 (0.52–7.80)	1.77 (0.46–6.85) ¶
	Yes	23.3%	22.3%	1.94 (0.50–7.50)	1.08 (0.27–4.33) ¶
	Female	43.3%	36.5%	2.40 (0.68–8.44)	1.62 (0.45–5.81) ¶
MDS-UPDRS Part III		0.0 (0.0-2.8)	0.0 (0.0–1.0)	1.21 (1.05–1.40)	1.14 (0.98–1.33)
	Excluding Tremor	0.0 (0.0–2.0)	0.0 (0.0–0.0)	1.32 (1.10–1.59)	1.32 (1.07–1.63)

BMI, body mass index; DEB, dream enactment behavior; K-GDS, Korean version of the Geriatric Depression Scale; MMSE, Mini-Mental State Examination; MoCA, Montreal Cognitive Assessment; PSQI, Pittsburgh Sleep Quality Index; RLS, restless legs syndrome; SCOPA-AUT, Scale for Outcomes in Parkinson’s disease for Autonomic symptoms; * age and sex are only corrected for sex and age, respectively. † Olfactory loss is defined as 1.5 standard deviation or less on a KVSS 1 or 2 test. ‡ Daily coffee consumption represents the average daily intake (cups per day), with coffee use defined as “yes” if daily coffee consumption > 0. § Elementary school graduate or lacking formal education. ‖ For each SCOPA-AUT subcategory, the subcategory is categorized as Yes if there is a score of 2 or more in that corresponding item; No if all are 0; otherwise, Borderline. ¶ SCOPA-AUT Erectile Dysfunction is not adjusted for sex.

**Table 2:** Participant characteristics of cognition- and motor-first subtypes.

Variable		Cognition-First *n* = 9	Motor-First *n* = 21	*p*-Value
**Age**		**75.0 (72.0–77.0)**	**67.0 (64.0–71.0)**	**0.02**
Sex, % male		55.6%	57.1%	1.00
Height, cm		160.0 (157.0–165.0)	165.0 (156.7–167.0)	0.62
Weight, kg		60.0 (58.4–67.0)	65.4 (56.0–67.7)	0.87
BMI, kg/m^2^		23.6 (22.8–24.6)	23.2 (22.2–24.7)	0.80
RLS		0.0%	9.5%	0.87
Diabetes		11.1%	19.0%	1.00
Melatonin		77.8%	47.6%	0.26
Antidepressants		22.2%	28.6%	1.00
Alcohol use				0.74
	Non-drinker	66.7%	66.7%	
	1–2 times/month	0.0%	14.3%	
	1–2 times/week	22.2%	19.0%	
	3–4 times/week	11.1%	0.0%	
	Daily	0.0%	0.0%	
Smoking				
	Never	77.8%	61.9%	0.67
	Current	11.1%	4.8%	1.00
	Former	11.1%	33.3%	0.42
Pesticide exposure		33.3%	14.3%	0.49
Solvent exposure		44.4%	23.8%	0.49
Olfactory loss *		55.6%	52.4%	1.00
Injury				0.71
	None	66.7%	61.9%	
	Minor	33.3%	28.6%	
	Major	0.0%	9.5%	
Coffee use †		77.8%	57.1%	0.51
Daily coffee consumption (cups) †		2.0 (1.0–2.0)	1.0 (0.0–2.0)	0.26
DEB frequency		3.0 (3.0–4.0)	3.0 (0.5–7.0)	0.66
Low education ‡		33.3%	23.8%	0.93
Education				0.35
	No education	11.1%	0.0%	
	Elementary school (6 years)	22.2%	23.8%	
	Middle school (9 years)	22.2%	19.0%	
	High school (12 years)	33.3%	23.8%	
	Bachelor’s degree (16 years)	0.0%	28.6%	
	Above Bachelor’s degree (18 years)	11.1%	4.8%	
First-degree relative with PD		11.1%	4.8%	1.00
RBDQ-KR		51.0 (30.0–62.0)	49.0 (40.0–54.0)	1.00
	RBDQ–KR Factor 1	8.0 (4.0–19.0)	12.0 (9.0–19.0)	0.43
	RBDQ–KR Factor 2	34.0 (22.0–43.0)	36.0 (30.0–37.0)	0.80
**MMSE**		**26.0 (23.0–26.0)**	**28.0 (26.0–29.0)**	**0.01**
**MoCA**		**23.0 (19.0–24.0)**	**26.0 (24.0–28.0)**	**0.03**
Epworth Sleepiness Scale		5.0 (3.0–7.0)	5.0 (4.0–7.0)	0.96
K-GDS		11.0 (4.0–15.0)	11.0 (7.0–17.0)	0.48
Insomnia Severity Index		7.0 (2.0–8.0)	7.0 (6.0–13.0)	0.52
PSQI		4.0 (3.0–4.0)	6.0 (4.0–9.0)	0.09
	**TST**	**8.4 (7.9–8.8)**	**7.0 (6.5–7.5)**	**0.03**
	C1	2.0 (1.0–3.0)	2.0 (1.0–2.0)	0.45
	C2	0.0 (0.0–0.0)	1.0 (0.0–1.0)	0.10
	C3	0.0 (0.0–1.0)	1.0 (0.0–1.0)	0.27
	C4	0.0 (0.0–0.0)	0.0 (0.0–1.0)	0.24
	C5	1.0 (1.0–1.0)	1.0 (1.0–2.0)	0.06
	C6	0.0 (0.0–0.0)	0.0 (0.0–1.0)	0.36
	C7	0.0 (0.0–1.0)	1.0 (0.0–1.0)	0.22
SCOPA-AUT total		13.0 (9.0–20.0)	12.0 (6.0–19.0)	0.95
SCOPA-AUT Orthostatic hypotension §				
	No	66.7%	52.4%	0.75
	Borderline	22.2%	42.9%	0.51
	Yes	11.1%	4.8%	1.00
SCOPA-AUT Constipation §				
	No	44.4%	19.0%	0.32
	Borderline	22.2%	57.1%	0.17
	Yes	33.3%	23.8%	0.93
SCOPA-AUT Urinary §				
	No	66.7%	52.4%	1.00
	Borderline	22.2%	42.9%	
	Yes	11.1%	4.8%	
SCOPA-AUT Erectile Dysfunction §				
	No	11.1%	9.5%	1.00
	Borderline	22.2%	23.8%	1.00
	Yes	22.2%	23.8%	1.00
	Female	44.4%	42.9%	1.00
MDS-UPDRS Part III		0.0 (0.0–1.0)	1.0 (0.0–3.0)	0.20
	Excluding Tremor	0.0 (0.0–1.0)	0.0 (0.0-2.0)	0.45
Conversion time		3.0 (2.0–4.0)	3.0 (2.0–3.0)	0.41

BMI, body mass index; DEB, dream enactment behavior; K-GDS, Korean version of the Geriatric Depression Scale; MMSE, Mini-Mental State Examination; MoCA, Montreal Cognitive Assessment; PSQI, Pittsburgh Sleep Quality Index; RLS, restless legs syndrome; SCOPA-AUT, Scale for Outcomes in Parkinson’s disease for Autonomic symptoms. * Olfactory loss is defined as 1.5 standard deviation or less on a KVSS 1 or 2 test. KVSS (Korean Version of Sniffin’ Sticks) is a standardized olfactory function test used to assess odor threshold, discrimination, and identification. † Daily coffee consumption represents the average daily intake (cups per day), with coffee use defined as “yes” if daily coffee consumption > 0. ‡ Elementary school graduate or lacking formal education. § For each SCOPA-AUT subcategory, the subcategory is categorized as Yes if there is a score of 2 or more in that corresponding item; No if all are 0; otherwise, Bold values indicate statistically significant results (*p* < 0.05).

## Data Availability

The data that support the findings of this study are available from the corresponding author upon reasonable request. Due to privacy and ethical restrictions, the data are not publicly available.

## Supplementary Materials

The following supporting information can be downloaded at: https://www.mdpi.com/article/10.3390/clockssleep7020019/s1, Figure S1: Kaplan–Meier estimate of the cohort; Figure S2: evaluation of phenoconversion time prediction models using different feature selection methods based on Uno’s concordance index; Figure S3: evaluation of phenoconversion time prediction models using different feature selection methods based on the integrated Brier score; Figure S4: beeswarm plot and bar plot of SHAP values for the time prediction model; Figure S5: SHAP dependence plots for selected features in the phenoconversion time prediction model; Figure S6: performance of classifiers for phenoconversion subtype prediction using the mRMR feature set; Figure S7: evaluation of classifiers for phenoconversion subtype prediction using univariable features; Figure S8: explanation of the phenoconversion subtype prediction model using SHAP; Figure S9: SHAP dependence plots for selected features in the phenoconversion subtype prediction model; Figure S10: web application for phenoconversion prediction in isolated REM sleep behavior disorder (iRBD); Figure S11: overview of the study design and methodology; Table S1: multivariate Cox regression analysis of predictors of phenoconversion in iRBD; Table S2: features selected by different feature selection methods in survival analysis; Table S3: Maximal Information Coefficient (MIC) values for differentiating motor-first and cognition-first groups; Table S4: performance of classifiers developed with the mRMR feature set.

## Abbreviations

The following abbreviations are used in this manuscript:

C-index	concordance index
CV	cross-validation
DLB	dementia with Lewy bodies
EEG	electroencephalography
ESS	Epworth Sleepiness Scale
GDS	Geriatric Depression Scale
HR	hazard ratio
IBS	integrated Brier score
iRBD	isolated REM sleep behavior disorder
ISI	Insomnia Severity Index
LPOCV	leave-pair-out cross-validation
MCC	Matthews correlation coefficient
MDS-UPDRS III	Movement Disorders Society–Unified Parkinson’s Disease Rating Scale part III
ML	machine learning
MMSE	Mini-Mental State Examination
MoCA	Montreal Cognitive Assessment
MSA	multiple system atrophy
PD	Parkinson’s disease
PSG	polysomnography
PSQI	Pittsburgh Sleep Quality Index
RBDQ-KR	Korean version of the REM Sleep Behavior Disorder Questionnaire–Hong Kong
RFE	recursive feature elimination
SCOPA-AUT	Scales for Outcomes in Parkinson’s Disease-Autonomic Dysfunction
SHAP	SHapley Additive exPlanations
SMOTE	Synthetic Minority Over-sampling Technique
XGBSE-KN	extreme gradient boosting survival embeddings–Kaplan neighbors
