# Supplementary material for: Predicting Phenoconversion in Isolated RBD: Machine Learning and Explainable AI Approach
Source: Clocks Sleep. 2025 Apr 11;7(2):19. doi: 10.3390/clockssleep7020019 (PMC12015906; doi:10.3390/clockssleep7020019)

Supplementary Figure 1. Kaplan-Meier Estimate of the Cohort

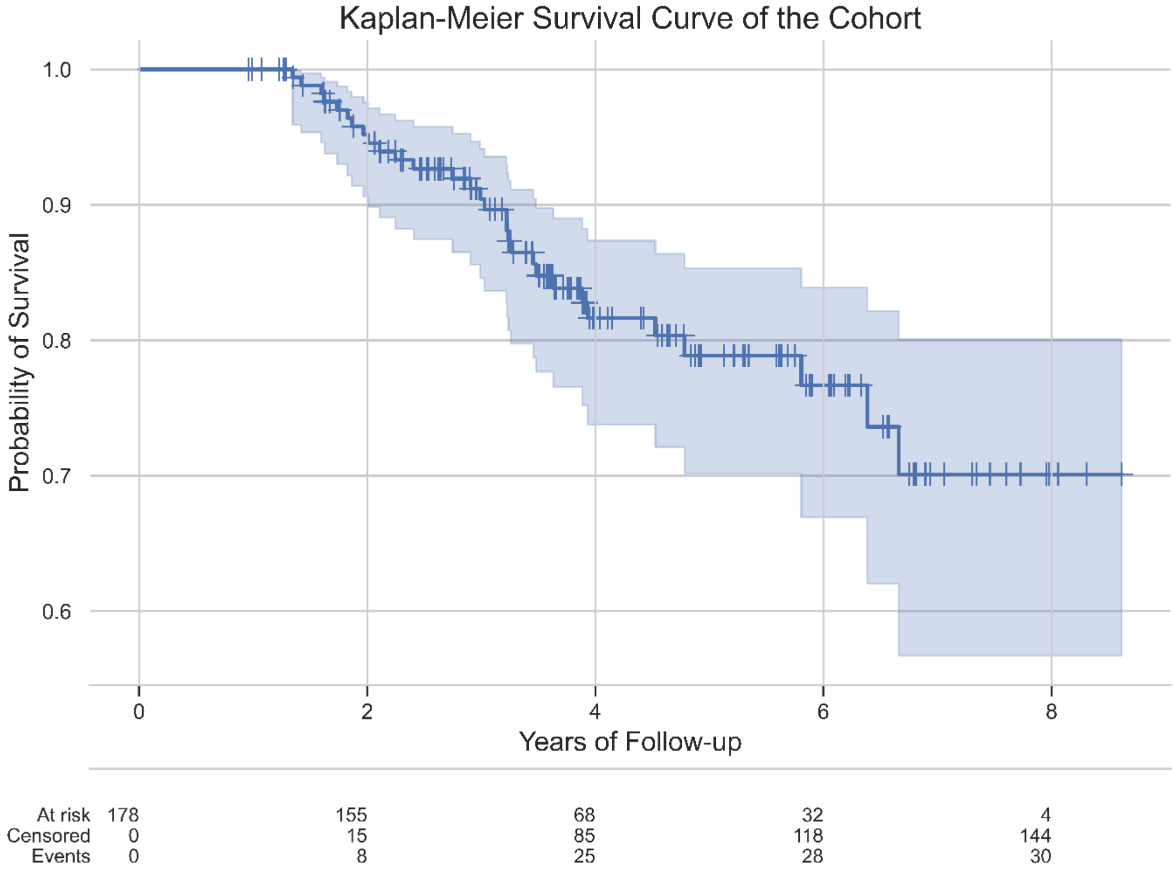

Supplementary Figure 2. Evaluation of phenoconversion time prediction models using different feature selection methods based on Uno's Concordance Index

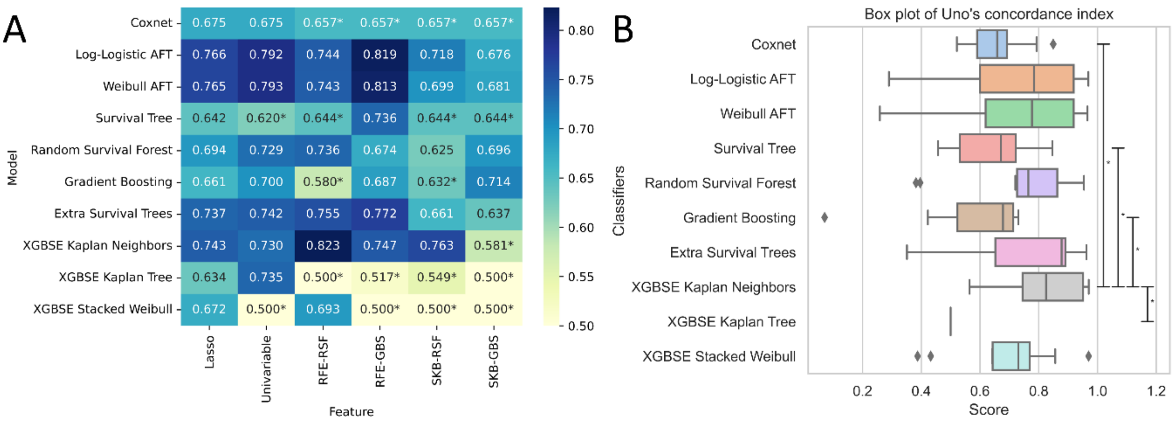

(A) Uno's concordance index values by model and feature selection method. (B) Uno's concordance index values for models utilizing the RFE-RSF (Recursive Feature Elimination-Random Survival Forests) feature set.

Supplementary Figure 3. Evaluation of phenoconversion time prediction models using different feature selection methods based on the Integrated Brier Score.

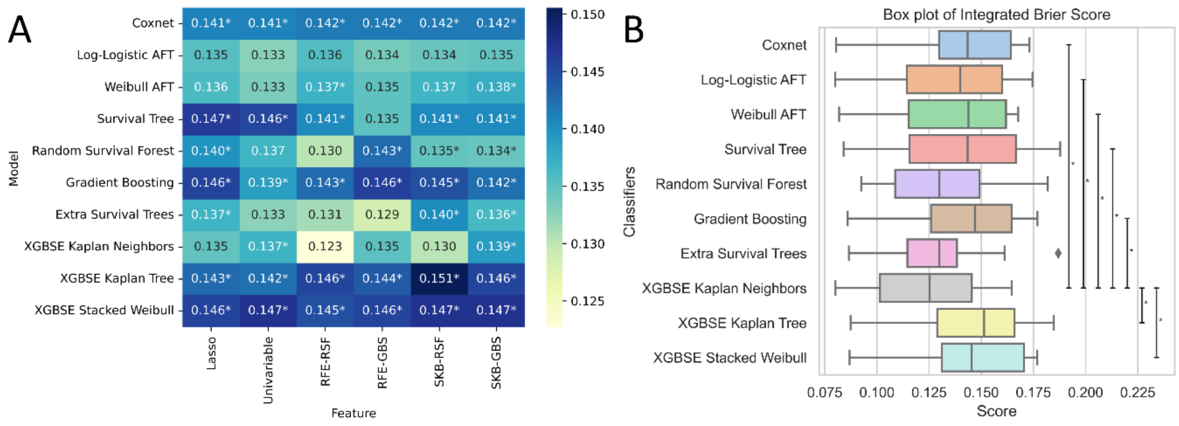

(A) Integrated Brier scores by model and feature selection method. (B) Integrated Brier scores of models using the RFE-RSF (Recursive Feature Elimination-Random Survival Forests) feature set.

Supplementary Figure 4. Two Plots for a Time Prediction Model: (A) Beeswarm Plot of SHAP Values for Each Feature (B) Bar Plot of Mean Absolute SHAP Values, Sorting Features by Importance

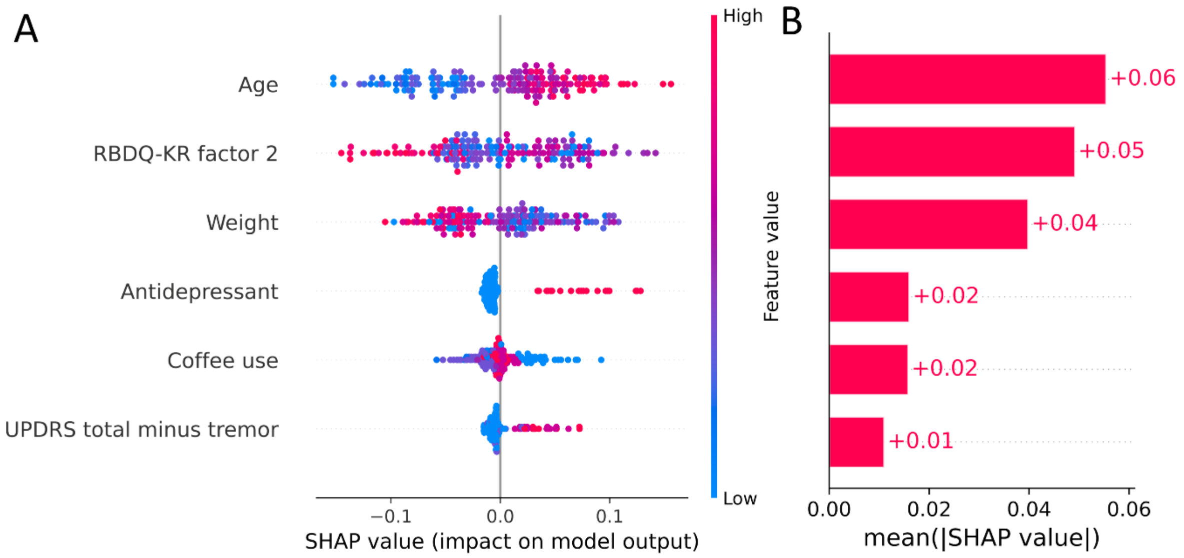

Supplementary Figure 5. SHAP dependence plots for selected features in the phenoconversion time prediction model.

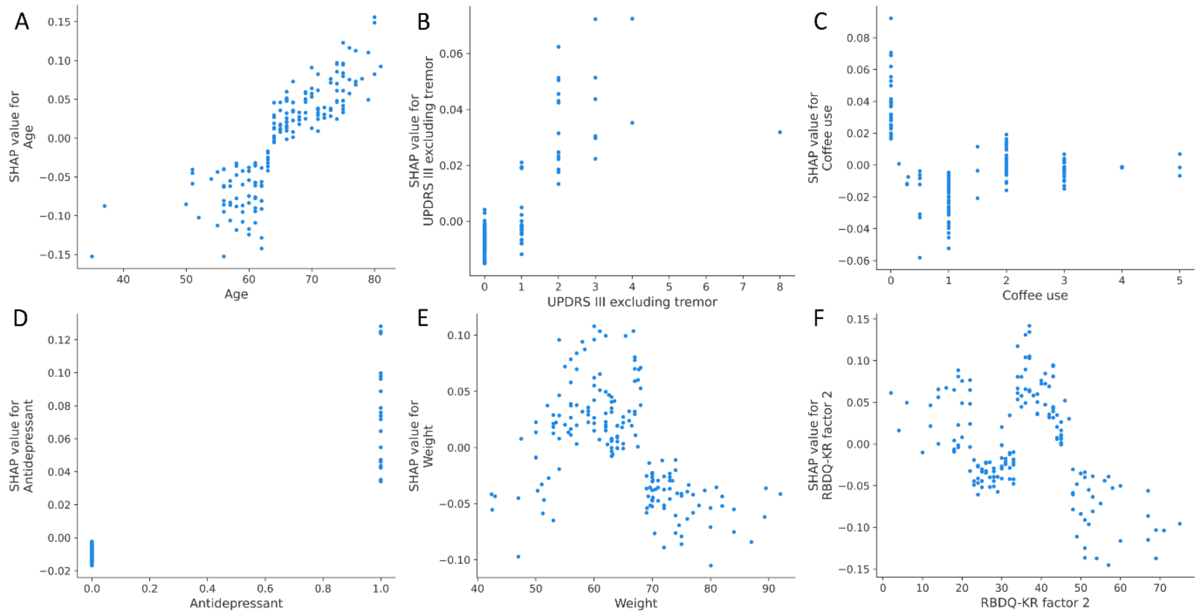

(A) Age. (B) Unified Parkinson's Disease Rating Scale (UPDRS) Part III excluding tremor. (C) Coffee use. (D) Antidepressant use. (E) Weight. (F) REM Sleep Behavior Disorder Questionnaire-Korea (RBDQ-KR) factor 2.

Supplementary Figure 6. Performance of classifiers for phenoconversion subtype prediction using the minimum-redundancy-maximum-relevance (mRMR) feature set.

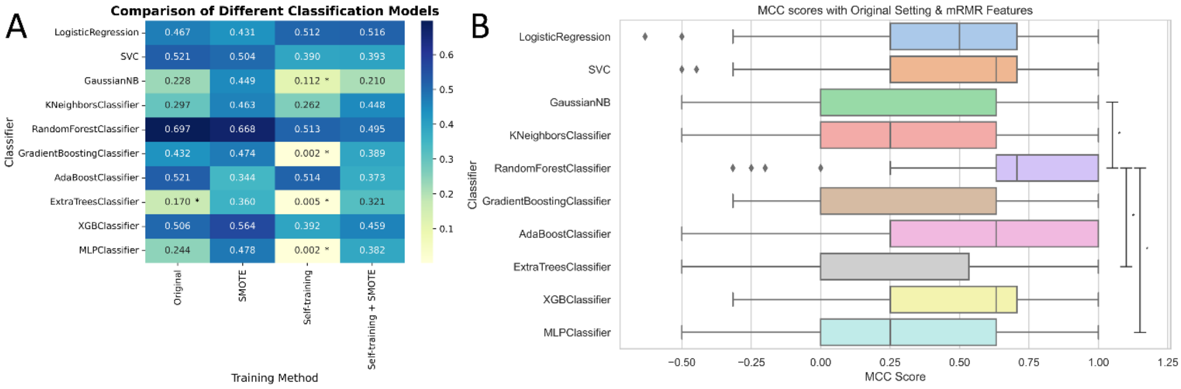

(A) Matthews Correlation Coefficient scores of classifiers using mRMR features. (B) Matthews Correlation Coefficient scores of models using original settings and the mRMR feature set.

Supplementary Figure 7. Evaluation of classifiers for phenoconversion subtype prediction using univariable features.

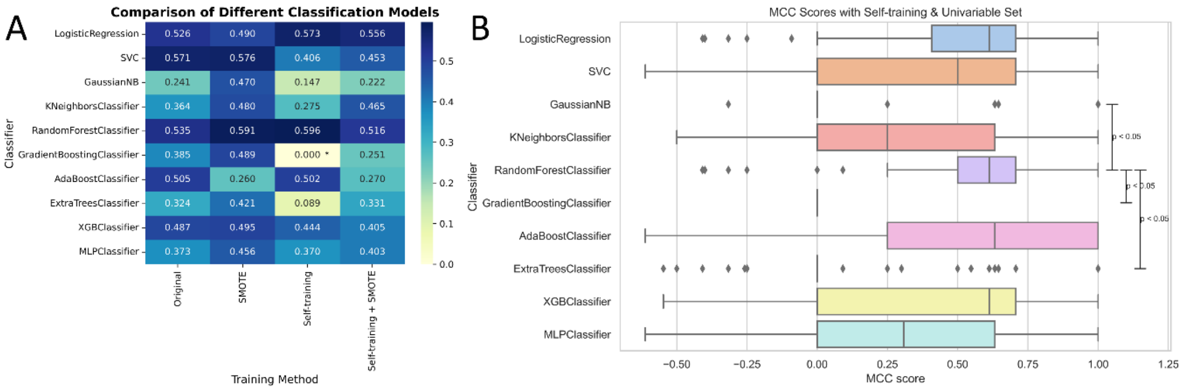

(A) MCC scores of classifiers using univariable features. (B) MCC scores of models using Self-training and the univariable feature set.

Supplementary Figure 8. Explanation of the phenoconversion subtype prediction model using SHapley Additive exPlanations (SHAP).

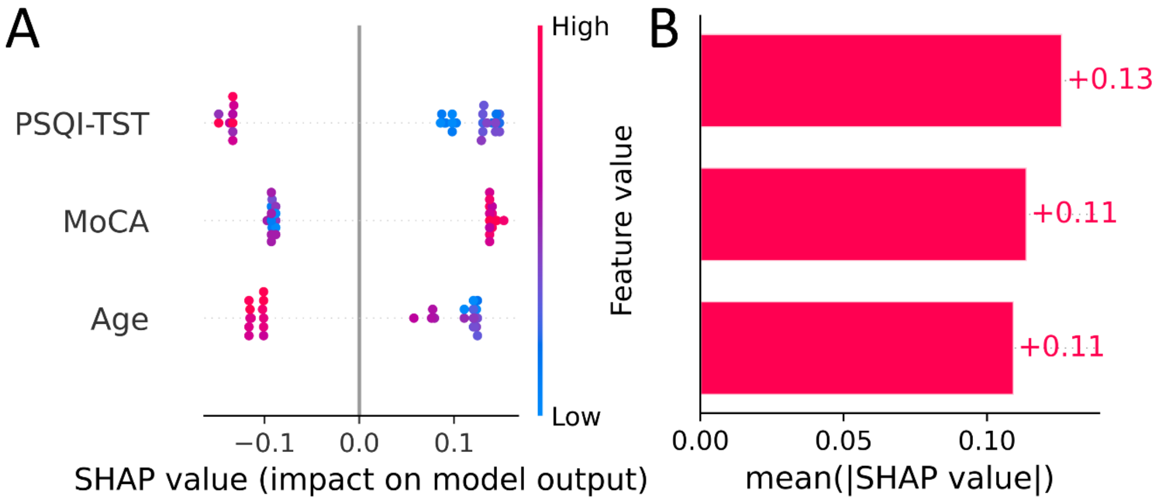

(A) Beeswarm plot of SHAP values for each feature. (B) Bar plot of mean absolute SHAP values, sorting features by importance.

Supplementary Figure 9. SHAP dependence plots for selected features in the phenoconversion subtype prediction model.

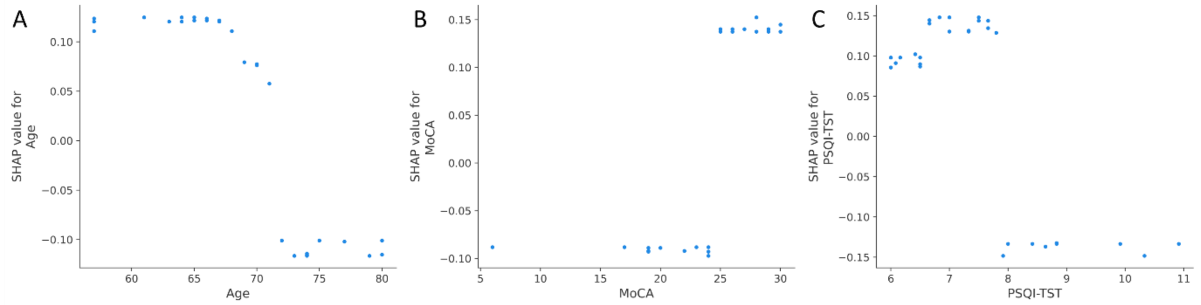

(A) Age. (B) Montreal Cognitive Assessment (MoCA) score. (C) Pittsburgh Sleep Quality Index-Total Sleep Time (PSQI-TST).

Supplementary Figure 10. Web application for phenoconversion prediction in isolated REM sleep behavior disorder (iRBD).

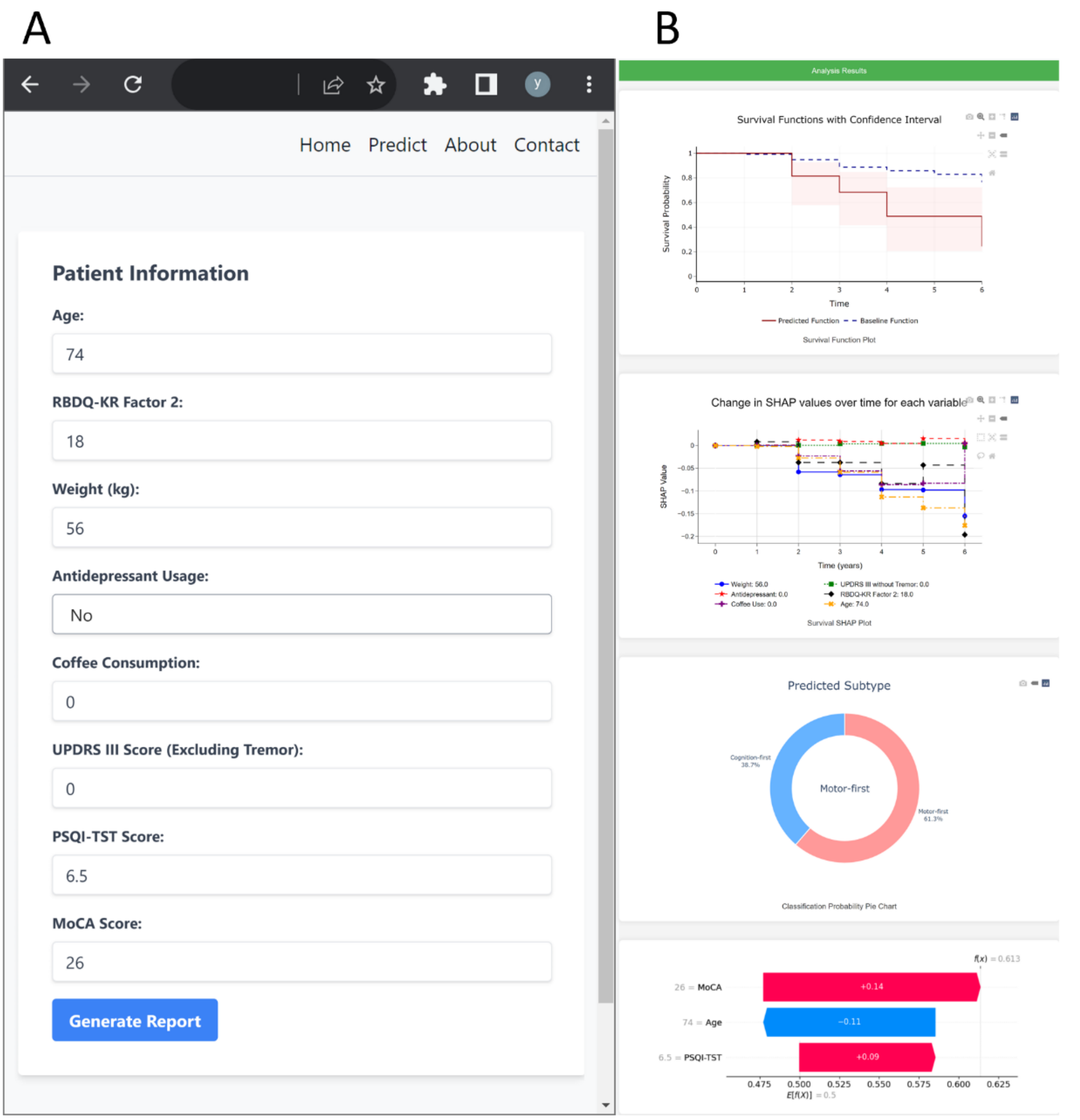

(A) Clinical indicator input page. (B) Example of a generated report based on the input data.

Supplementary Figure 11. Overview of the study design and methodology

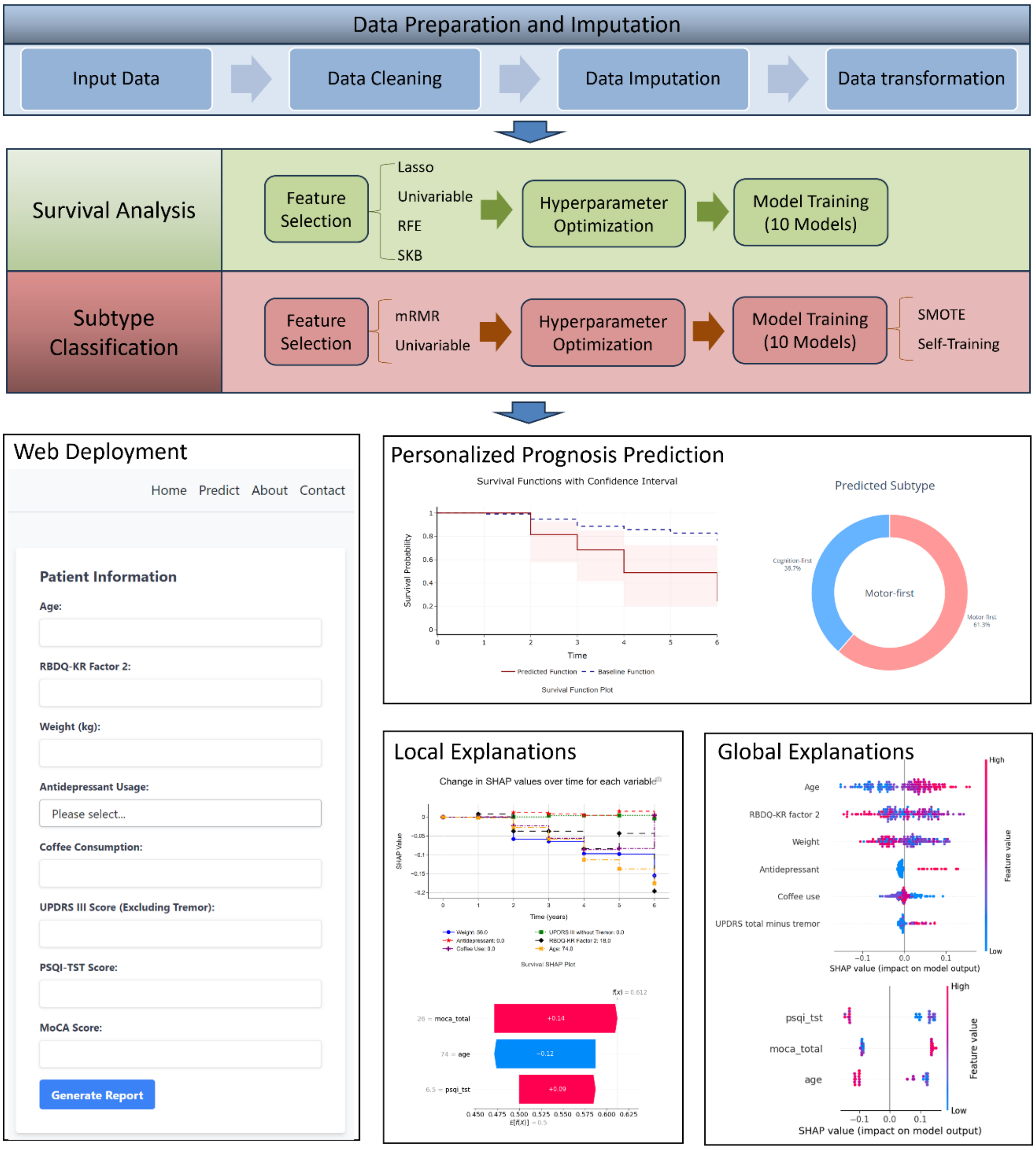

Supplement: Supplementary file 1 [file clockssleep-07-00019-s001.zip › Supplemental Figures.pdf]
