# Supplementary material for: Predicting Phenoconversion in Isolated RBD: Machine Learning and Explainable AI Approach
Source: Clocks Sleep. 2025 Apr 11;7(2):19. doi: 10.3390/clockssleep7020019 (PMC12015906; doi:10.3390/clockssleep7020019)
Supplement: Supplementary file 1 [file clockssleep-07-00019-s001.zip › Supplemental Tables.pdf]

Supplementary Table 1. Multivariate Cox Regression Analysis of Predictors of Phenoconversion in iRBD

Variable	Hazard Ratio (HR)	95% Confidence Interval (CI)	P-value
Education	1.05	0.66-1.68	0.83
Antidepressant	1.3	0.95-1.77	0.1
Age	1.82	1.15-2.88	0.01
UPDRS Part III	1.03	0.65-1.61	0.91
Excluding tremor	1.2	0.87-1.65	0.27
Solvent exposure	1.42	0.99-2.02	0.06
Coffee use *	0.78	0.53-1.14	0.2
MMSE	0.84	0.52-1.35	0.47
MoCA	1.08	0.71-1.62	0.72
PSQI-C3	0.59	0.37-0.95	0.03
PSQI-TST	0.95	0.54-1.67	0.85

MMSE, Mini Mental State Examination; MoCA, Montreal Cognitive Assessment; PSQI, Pittsburgh Sleep Quality Index; TST, Total Sleep Time.

* Coffee use is defined as "yes" if daily coffee consumption >0.

Supplementary Table 2. Features selected by different feature selection methods in survival analysis.

Method	Selected Features
Univariable	Age, Antidepressant, Coffee use, Education, MMSE, MoCA, PSQI-C3, PSQI-TST, Solvent exposure, UPDRS III, UPDRS III excluding tremor
Lasso	Age, Antidepressant, UPDRS III excluding tremor
RFE-RSF	Age, Antidepressant, Daily coffee consumption, RBDQ-KR factor 2, UPDRS III excluding tremor, Weight
RFE-GBS	Age, Antidepressant, BMI, Daily coffee consumption, K-GDS, PSQI-C4, PSQI-TST, RBDQ-KR factor 2, UPDRS III excluding tremor, Weight
SKB-RSF	Age, Antidepressant, Coffee use, Daily coffee consumption, MoCA, RBDQ-KR factor 2, Solvent exposure
SKB-GBS	Age, Antidepressant, Daily coffee consumption, RBDQ-KR Factor 2

GBS, Gradient Boosting Survival Analysis; K-GDS, Korean version of the Geriatric Depression Scale; MMSE, Mini-Mental State Examination; MoCA, Montreal Cognitive Assessment; PSQI-C3, A component of the Pittsburgh Sleep Quality Index related to sleep duration; PSQI-TST, Total Sleep Time in the Pittsburgh Sleep Quality Index; RBDQ-KR factor 2, A factor within the REM Sleep Behavior Disorder Questionnaire - Korea version, assessing behavioral symptoms; RFE, Recursive Feature Elimination; RSF, Random Survival Forest; UPDRS III, Unified Parkinson's Disease Rating Scale Part III; UPDRS III excluding tremor, A metric within UPDRS III excluding tremor assessment.

Note: Daily coffee consumption represents the average daily intake (cups per day), with Coffee use defined as "yes" if daily coffee consumption >0.

Supplementary Table 3. Maximal Information Coefficient (MIC) Values for Differentiating Motor-first and Cognition-first Groups

Variable	MIC	P-value
**PSQI-TST**	**0.435689**	**0.00570**
**MMSE**	**0.293764**	**0.02770**
MoCA	0.281291	0.05630
Age	0.256128	0.14465
RBDQ-KR Factor 2	0.193507	0.38760
ISI	0.187807	0.44660
GDS-K	0.186189	0.42890
PSQI-Total	0.174716	0.41690
Weight	0.160395	0.68530

GDS-K, Geriatric Depression Scale-Korean version; ISI, Insomnia Severity Index; MIC, Maximal Information Coefficient; MMSE, Mini-Mental State Examination; MoCA, Montreal Cognitive Assessment; PSQI-Total, Pittsburgh Sleep Quality Index Total Score; PSQI-TST, Pittsburgh Sleep Quality Index - Total Sleep Time; RBDQ-KR Factor 2, REM Sleep Behavior Disorder Questionnaire-Korea, Factor 2.

Supplementary Table 4. Performance of Classifiers developed with the mRMR feature set.

Classifier	MCC	Macro F1	Accuracy	Macro Precision	Macro Recall	Balanced Accuracy	Log Loss	Cohen's Kappa	ROC AUC	PR AUC	LPOCV AUC
RandomForestClassifier	**<u>0.697</u>**	**<u>0.825</u>**	**<u>0.853</u>**	**<u>0.850</u>**	**<u>0.851</u>**	**<u>0.851</u>**	0.500	**<u>0.665</u>**	**<u>0.876</u>**	0.799	**<u>0.881</u>**
SVC	**0.521**	0.733	0.775	**0.760**	0.762	0.762	0.615	0.489	0.727	0.724	0.804
AdaBoostClassifier	**0.521**	**0.737**	**0.791**	0.753	**0.763**	**0.763**	1.575	**0.500**	0.766	0.626	0.820
XGBClassifier	0.506	0.727	0.795	0.744	0.751	0.751	**<u>0.468</u>**	0.483	**0.860**	**0.824**	**0.865**
LogisticRegression	0.467	0.697	0.738	0.724	0.742	0.742	0.581	0.428	0.829	0.772	0.825
GradientBoostingClassifier	0.432	0.679	0.785	0.704	0.703	0.703	**0.489**	0.407	0.856	**<u>0.830</u>**	0.862
KNeighborsClassifier	0.297	0.613	0.737	0.625	0.639	0.639	2.708	0.280	0.742	0.697	0.733
MLPClassifier	0.244	0.576	0.713	0.580	0.618	0.618	0.546	0.226	0.820	0.759	0.820
GaussianNB	0.228	0.562	0.735	0.560	0.604	0.604	0.662	0.214	0.779	0.755	0.778
ExtraTreesClassifier	0.170	0.543	0.689	0.535	0.586	0.586	0.552	0.160	0.769	0.716	0.767

LPOCV AUC, Leave-Pair-Out Cross-Validation Area Under the Curve; Macro F1, macro-averaged F1 score; Macro Precision, macro-averaged precision; Macro Recall, macro-averaged recall; MCC, Matthews Correlation Coefficient; mRMR, minimum Redundancy Maximum Relevance; PR AUC, Area Under the Precision-Recall Curve; ROC AUC, Area Under the Receiver Operating Characteristic Curve. The classifiers are ordered by descending MCC score. All metrics are reported on the test set. For each metric, the highest value is highlighted in bold and underlined, while the second-highest value is shown in bold.
